# Data on assessment of groundwater quality for drinking and irrigation in rural area Sarpol-e Zahab city, Kermanshah province, Iran

**DOI:** 10.1016/j.dib.2017.12.061

**Published:** 2018-01-03

**Authors:** Hamed Soleimani, Abbas Abbasnia, Mahmood Yousefi, Ali Akbar Mohammadi, Fazlollah Changani Khorasgani

**Affiliations:** a Department of Environmental Health Engineering, School of Public Health, Tehran University of Medical Sciences, Tehran, Iran; b Department of Environmental Health Engineering, Neyshabur University of Medical Sciences, Neyshabur, Iran

**Keywords:** Groundwater quality index, Rural area, Sarpol-e Zahab, Iran

## Abstract

In present study 30 groundwater samples were collected from Sarpol-e Zahab area, Kermanshah province of Iran in order to assess the quality of groundwater in subjected area and determining its suitability for drinking and agricultural purposes. Also the variations in the quality levels of groundwater were compared over the years of 2015 and 2016. Statistical analyses including Spearman correlation coefficients and factor analysis display good correlation between physicochemical parameters (EC, TDS and TH) and Na^+^, Mg^2+^, Ca^2+^, Cl^−^ and ${\mathrm{SO}}_{4}^{2-}$ ionic constituents. Also in order to assess water quality for irrigation we used the United States Department of Agriculture (USDA) classification which is based on SAR for irrigation suitability assessment. In addition, the residual sodium carbonate (RSC), %Na, PI, KR, SSP, MH, EC characteristics were calculated for all samples and used for assessment of irrigation suitability. Based on these indicators, for every two years, the quality of water for agriculture is in good and excellent category. The Piper classification for hydro geochemical facies indicates that the water in the study area is of Ca-HCO_3_^−^ type. However, the study of water hardness shows that more than 80% of samples are in hard and very hard water class. Therefore, there is a need for decisions to refine and soften the water.

**Specifications Table**

**Table:** 

Subject area	Chemistry
More specific subject area	Describe narrower subject area
Type of data	Tables and figures
How data was acquired	Experiments have been done in two total categories of system tests and titrimetric tests including temporary and permanent hardness, calcium, magnesium and chloride. Also system tests including pH and electrical conductivity (EC) measured by pH meter device (pHwtw model) and Esi meter (wbw), respectively. The analysis of anions and cations of sulfate was also done by spectrophotometer Hatch (DR 5000 model) in water and wastewater laboratory of Kermanshah. Total hardness was determined by EDTA titrimetric method and TDS was measured gravimetrically.
Data format	Raw, Analyzed
Experimental factors	All water samples in polyethylene bottles were stored in a dark place at room temperature until the metals analysis
Experimental features	The mentioned parameters above, in abstract section, were analyzed according to the standards for water and wastewater treatment handbook.
Data source location	Sarpol-e Zahab, Kermanshah province, Iran
Data accessibility	Data are included in this article and supplement file excel

**Value of the data**

• Determination of the physical and chemical parameter including EC, pH, TDS, TH, Ca, Mg, CO_3_, HCO_3_, Na, K, Cl and SO_4_ in ground water was investigated in rural area, Sarpol-e Zahab city, Iran.
• Due to limited studies in the study area, the data of this study can help to better understand the quality of groundwater in the area and provide further studies.
• The result of data analysis shows that water in this area is suitable for agricultural according to calculated indices.

## 1 Data

**Fig. 1:**
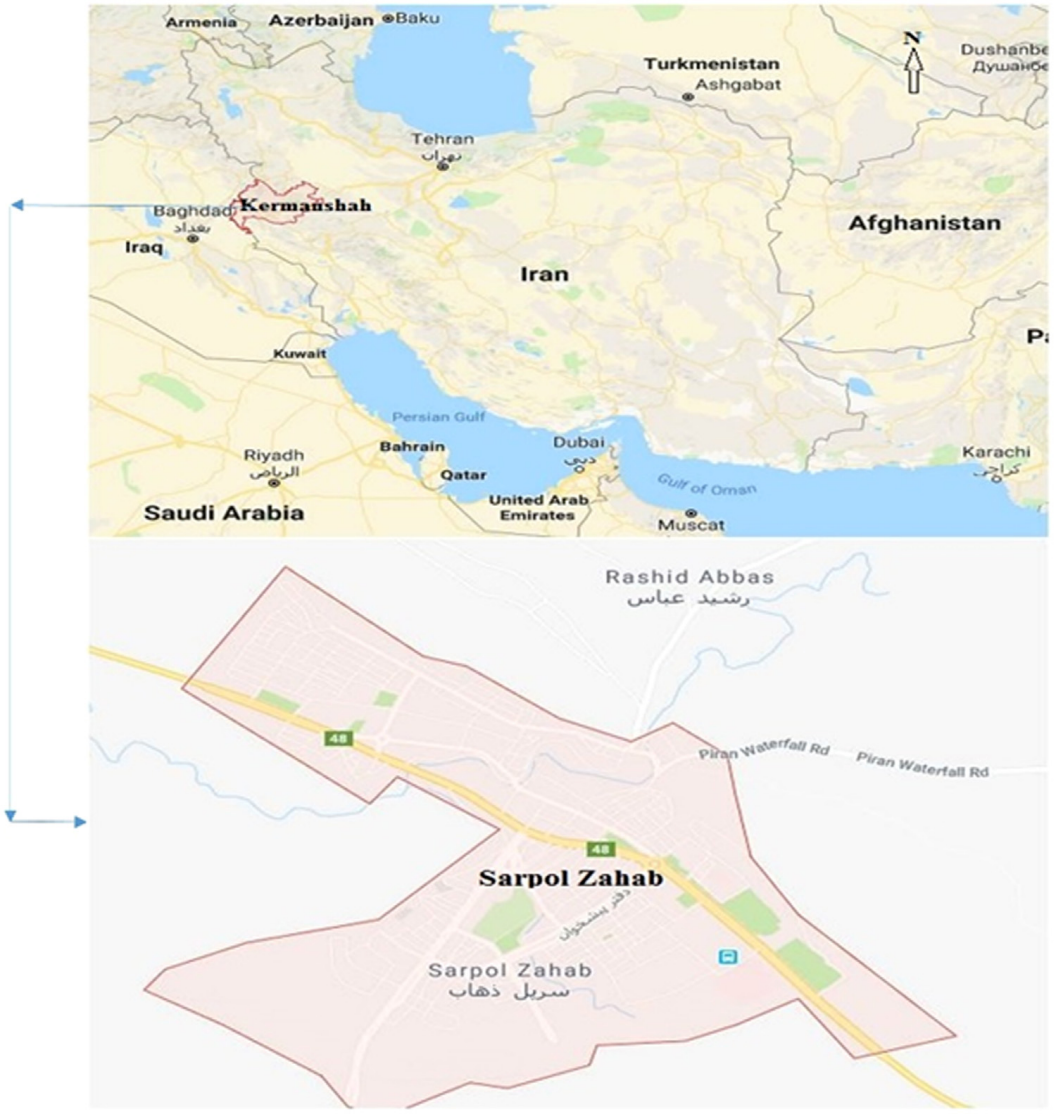
The map and location of sampling villages.

**Fig. 2:**
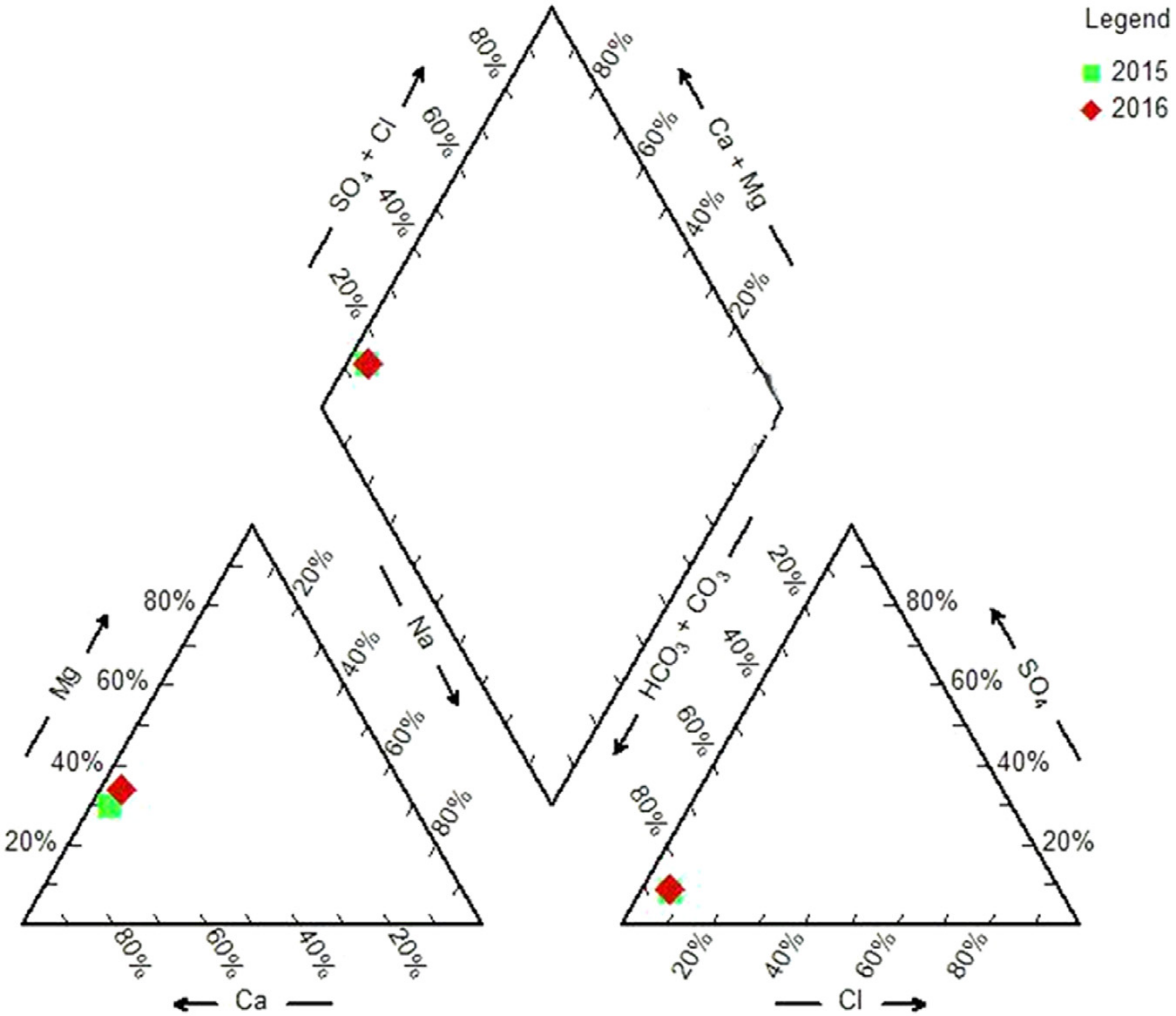
The Piper diagram indicates that the hydrochemical type of water.

The data presented here deal with monitoring of physical and chemical characteristics of groundwater including pH, EC, TDS, HCO_3_, CO_3_, SO_4_, Cl, Ca, Mg, and Na as well as in Sarpol-e Zahab city, Kermanshah, Iran. The study area and the sampling points are shown in Fig. 1. Also a summary of water quality characteristics are presented in Table 1, Table 2. Results of quality assessment of groundwater samples from rural area in city for drinking purpose (BIS standard) are presented in Table 3, Table 4 [1]. Also classification of groundwater samples for irrigation use on the basis of EC, SAR, RSC, KR, SSP, PI, MH, Na%, T.H are presented in Table 5. Finally, the Piper diagram indicates that the Hydrochemical type of water is of Ca-HCO_3_ type (Fig. 2) (Table 6, Table 7).

**Table 1:** Water level and physico-chemical analyses of groundwater samples of study area collected during 2015 year.

**Well**	**pH**	**Na**	**Mg**	**Ca**	**Cl**	**CO**_**3**_	**HCO**_**3**_	**SO**_**4**_	**TDS**	**EC**	**T.H**
**no**		**(mg/L)**	**(mg/L)**	**(mg/L)**	**(mg/L)**	**(mg/L)**	**(mg/L)**	**(mg/L)**	**(mg/l)**	**(μmhos/cm)**	**(mg/l)**
P1	7.33	5.75	21.78	90	17.75	0	335.5	25.44	430	672	315
P2	7.47	4.6	20.57	78	14.2	0	311.1	13.44	376	587	280
P3	7.48	4.6	16.94	76	10.65	0	292.8	13.44	354	553	260
P4	8.07	5.75	12.1	64	10.65	0	189.1	49.44	292	457	210
P5	7.19	25.07	18.15	90	28.4	0	335.5	36.96	465	715	300
P6	7.38	20.01	14.52	80	24.85	0	305	16.8	395	617	260
P7	8.03	4.6	20.57	58	14.2	0	244	18.24	316	493	230
P8	8.15	6.44	29.04	58	17.75	0	262.3	36.48	365	570	265
P9	7.7	7.36	18.15	90	17.75	0	305	38.4	412	644	300
P10	7.71	2.76	16.94	56	10.65	0	225.7	14.4	272	425	210
P11	7.55	8.97	33.88	92	17.75	0	408.7	27.36	519	798	370
P12	8.28	3.68	16.94	62	10.65	0	244	16.32	306	478	225
P13	7.62	2.76	18.15	54	10.65	0	225.7	14.4	283	442	210
P14	7.81	4.6	16.94	76	10.65	0	280.6	23.04	351	548	260
P15	8.04	3.68	19.36	54	10.65	0	231.8	16.32	295	461	215
P16	8.06	6.44	12.1	58	10.65	0	219.6	12.48	274	428	195
P17	7.71	1.38	15.73	52	7.1	0	213.5	11.52	265	414	195
P18	7.45	5.75	21.78	80	14.2	0	305	30.24	393	614	290
P19	7.68	4.6	16.94	72	10.65	0	280.6	13.44	342	534	250
P20	7.65	4.6	16.94	80	14.2	0	298.9	13.44	367	573	270
P21	7.97	5.75	25.41	76	14.2	0	305	35.04	401	626	295
P22	7.71	4.6	19.36	70	10.65	0	262.3	32.64	346	540	255
P23	7.35	11.73	33.88	100	24.85	0	408.7	42.72	550	846	390
P24	7.46	3.68	16.94	60	10.65	0	244	11.52	302	472	220
P25	7.66	2.76	18.15	50	7.1	0	225.7	9.6	269	420	200
P26	7.28	9.89	25.41	84	21.3	0	347.7	19.68	438	685	315
P27	8.18	2.07	10.89	66	10.65	0	225.7	12.96	284	444	210
P28	7.73	4.6	13.31	90	14.2	0	305	18.24	381	596	280
P29	7.51	3.68	15.73	68	7.1	0	268.4	11.52	319	499	235
P30	8	5.06	26.62	70	14.2	0	317.2	14.4	384	600	285
**Min**	7.2	1.4	10.9	50.0	7.1	0.0	189.1	9.6	265.0	414.0	195.0
**Max**	8.3	25.1	33.9	100.0	28.4	0.0	408.7	49.4	550.0	846.0	390.0
**Ave**	7.7	6.2	19.4	71.8	14.0	0.0	280.8	21.7	358.2	558.4	259.8
**SD**	0.30	5.00	5.77	13.95	5.35	0.00	54.12	11.02	73.08	111.47	49.30

**Table 2:** Water level and physico-chemical analyses of groundwater samples of study area collected during 2016 year.

**Well**	**pH**	**Na**	**Mg**	**Ca**	**Cl**	**CO**_**3**_	**HCO**_**3**_	**SO**_**4**_	**TDS**	**EC**	**T.H**
**no**		**(mg/L)**	**(mg/L)**	**(mg/L)**	**(mg/L)**	**(mg/L)**	**(mg/L)**	**(mg/L)**	**(mg/l)**	**(μ mhos/cm)**	**(mg/l)**
P1	7.3	14.95	42.35	104	31.95	0	378.2	107.04	614	944	435
P2	7.58	3.68	24.2	60	10.65	0	274.5	16.32	336	525	250
P3	7.63	5.75	12.1	60	10.65	0	219.6	15.84	281	439	200
P4	7.74	2.76	16.94	54	10.65	0	219.6	14.4	276	432	205
P5	7.27	25.07	22.99	80	24.85	0	359.9	17.76	463	712	295
P6	7.54	3.68	24.2	56	10.65	0	262.3	16.32	323	505	240
P7	7.54	4.6	25.41	74	10.65	0	323.3	18.24	388	607	290
P8	7.56	7.36	25.41	74	14.2	0	329.4	14.4	401	627	290
P9	7.83	2.76	15.73	54	10.65	0	213.5	14.4	268	418	200
P10	7.29	7.36	24.2	82	17.75	0	341.6	14.4	420	656	305
P11	7.84	7.36	10.89	60	10.65	0	219.6	14.4	275	430	195
P12	7.74	8.97	20.57	76	10.65	0	305	27.36	383	598	275
P13	7.42	5.75	25.41	74	17.75	0	317.2	15.84	392	612	290
P14	7.63	4.6	22.99	70	10.65	0	298.9	18.24	364	569	270
P15	7.46	5.75	24.2	68	17.75	0	292.8	15.84	372	581	270
P16	7.58	4.6	22.99	80	10.65	0	329.4	18.24	397	620	295
P17	7.56	7.36	24.2	78	14.2	0	335.5	14.4	405	633	295
P18	7.53	8.97	18.15	84	17.75	0	317.2	17.76	398	622	285
P19	7.34	3.68	21.78	60	10.65	0	262.3	16.32	324	506	240
P20	7.87	2.76	24.2	48	10.65	0	237.9	14.4	274	428	220
P21	7.34	4.6	19.36	70	10.65	0	280.6	18.24	344	538	255
P22	7.62	7.36	18.15	48	10.65	0	219.6	14.4	279	436	195
P23	7.59	5.75	15.73	58	10.65	0	231.8	15.84	292	457	210
P24	7.57	14.95	38.72	110	31.95	0	396.5	92.64	616	497	435
P25	7.06	6.44	27.83	76	10.65	0	335.5	26.88	414	647	305
P26	7.52	2.76	20.57	48	10.65	0	219.6	14.4	278	435	205
P27	7.33	4.6	25.41	50	10.65	0	244	23.04	312	487	230
P28	7.23	9.89	36.3	96	17.75	0	451.4	14.88	541	833	390
P29	7.26	8.05	29.04	88	21.3	0	305	59.04	470	723	340
P30	7.21	5.75	29.04	80	10.65	0	353.8	25.44	431	673	320
**Min**	7.1	2.8	10.9	48.0	10.7	0.0	213.5	14.4	268.0	418.0	195.0
**Max**	7.9	25.1	42.35	110.0	32.0	0.0	451.4	107.0	616.0	944.0	435.0
**Ave**	7.5	6.9	23.6	70.7	14.3	0.0	295.9	24.2	377.7	573.0	274.3
**SD**	0.20	4.60	6.94	16.27	6.15	0.00	60.70	22.30	94.10	126.54	64.62

**Table 3:** Calculation of RSC, PI, KR, MH, Na%, SAR and SSP of groundwater for 2015and 2016 years.

**Well**	**2015 Year**						**2016 Year**							
**ID**	**RSC**	**PI**	**KR**	**MH**	**Na%**	**SAR**	**SSP**	**RSC**	**PI**	**KR**	**MH**	**Na%**	**SAR**	**SSP**
P1	− 0.80	39.62	0.04	28.57	3.82	0.14	3.82	− 2.5	33.58	0.07	40.23	6.95	0.31	6.95
P2	− 0.50	42.38	0.04	30.36	3.45	0.12	3.45	− 0.5	44.21	0.03	40.00	3.10	0.10	3.10
P3	− 0.40	44.28	0.04	26.92	3.70	0.12	3.70	− 0.4	50.53	0.06	25.00	5.88	0.18	5.88
P4	− 1.10	45.18	0.06	23.81	5.62	0.17	5.62	− 0.5	47.80	0.03	34.15	2.84	0.08	2.84
P5	− 0.50	48.45	0.18	25.00	15.37	0.63	15.37	0	50.34	0.18	32.20	15.59	0.63	15.59
P6	− 0.20	51.17	0.17	23.08	14.33	0.54	14.33	− 0.5	45.03	0.03	41.67	3.23	0.10	3.23
P7	− 0.60	45.83	0.04	36.96	4.17	0.13	4.17	− 0.5	41.70	0.03	36.21	3.33	0.12	3.33
P8	− 1.00	42.18	0.05	45.28	5.02	0.17	5.02	− 0.4	43.20	0.06	36.21	5.23	0.19	5.23
P9	− 1.00	40.44	0.05	25.00	5.06	0.18	5.06	− 0.5	48.32	0.03	32.50	2.91	0.08	2.91
P10	− 0.50	47.30	0.03	33.33	2.78	0.08	2.78	− 0.5	41.84	0.05	32.79	4.98	0.18	4.98
P11	− 0.70	38.23	0.05	37.84	5.01	0.20	5.01	− 0.3	52.54	0.08	23.08	7.58	0.23	7.58
P12	− 0.50	46.35	0.04	31.11	3.43	0.11	3.43	− 0.5	44.59	0.07	30.91	6.62	0.24	6.62
P13	− 0.50	47.30	0.03	35.71	2.78	0.08	2.78	− 0.6	41.82	0.04	36.21	4.13	0.15	4.13
P14	− 0.60	43.42	0.04	26.92	3.70	0.12	3.70	− 0.5	43.10	0.04	35.19	3.57	0.12	3.57
P15	− 0.50	47.30	0.04	37.21	3.59	0.11	3.59	− 0.6	43.20	0.05	37.04	4.42	0.15	4.42
P16	− 0.30	52.09	0.07	25.64	6.70	0.20	6.70	− 0.5	41.37	0.03	32.20	3.28	0.12	3.28
P17	− 0.40	48.76	0.02	33.33	1.52	0.04	1.52	− 0.4	42.85	0.05	33.90	5.14	0.19	5.14
P18	− 0.80	41.09	0.04	31.03	4.13	0.15	4.13	− 0.5	43.85	0.07	26.32	6.40	0.23	6.40
P19	− 0.40	45.09	0.04	28.00	3.85	0.13	3.85	− 0.5	45.03	0.03	37.50	3.23	0.10	3.23
P20	− 0.50	43.10	0.04	25.93	3.57	0.12	3.57	− 0.5	46.35	0.03	45.45	2.65	0.08	2.65
P21	− 0.90	40.42	0.04	35.59	4.07	0.15	4.07	− 0.5	44.24	0.04	31.37	3.77	0.13	3.77
P22	− 0.80	42.90	0.04	31.37	3.77	0.13	3.77	− 0.3	52.54	0.08	38.46	7.58	0.23	7.58
P23	− 1.10	37.29	0.07	35.90	6.14	0.26	6.14	− 0.4	49.42	0.06	30.95	5.62	0.17	5.62
P24	− 0.40	47.37	0.04	31.82	3.51	0.11	3.51	− 2.2	34.22	0.07	36.78	6.95	0.31	6.95
P25	− 0.30	49.60	0.03	37.50	2.91	0.08	2.91	− 0.6	41.15	0.05	37.70	4.39	0.16	4.39
P26	− 0.60	41.86	0.07	33.33	6.39	0.24	6.39	− 0.5	47.80	0.03	41.46	2.84	0.08	2.84
P27	− 0.50	46.94	0.02	21.43	2.10	0.06	2.10	− 0.6	45.83	0.04	45.65	4.17	0.13	4.17
P28	− 0.60	42.00	0.04	19.64	3.45	0.12	3.45	− 0.4	38.28	0.06	38.46	5.22	0.22	5.22
P29	− 0.30	46.45	0.03	27.66	3.29	0.10	3.29	− 1.8	36.17	0.05	35.29	4.90	0.19	4.90
P30	− 0.50	42.24	0.04	38.60	3.72	0.13	3.72	− 0.6	39.97	0.04	37.50	3.76	0.14	3.76
**Min**	− 1.10	37.29	0.02	19.64	1.52	0.04	1.52	− 2.50	33.58	0.03	23.08	2.65	0.08	2.65
**Max**	− 0.20	52.09	0.18	45.28	15.37	0.63	15.37	0.00	52.54	0.18	45.65	15.59	0.63	15.59
**Ave**	− 0.59	44.56	0.05	30.80	4.70	0.16	4.70	− 0.64	44.03	0.05	35.41	5.01	0.18	5.01
**SD**	0.24	3.73	0.04	5.96	3.00	0.12	3.00	0.54	4.79	0.03	5.27	2.50	0.11	2.50

**Table 4:** Quality of ground water sample samples from rural area in Sarpol-e Zahab city for drinking purpose (BIS standard) [2].

**Parameter**	**Desirable limit**	**2015 Year samples (%)**		**2016 Year samples (%)**	
		**Within limits**	**Exceed limits**	**Within limits**	**Exceed limits**
**pH**	6.5–8.5	100	0	100	0
**EC**	300 (μmhos/cm)	0	100	0	100
**TDS**	500 (mg/L)	93.3	6.7	90	10
**Total hardness**	200 (mg/L)	13.4	86.6	20	80
**SO4**	200 (mg/L)	100	0	100	0
**Cl**	250 (mg/L)	100	0	100	0
**Ca**	75 (mg/L)	53.3	46.7	60	40
**Mg**	30 (mg/L)	93.3	6.7	90	10
**Na**	200 (mg/L)	100	0	100	0

**Table 5:** Classification of groundwater sample for irrigation use on the basic of EC, SAR, RSC, KR, SSP, PI, MH, Na%, T.H [2].

**Parameters**	**Range**	**Water class**	**Samples(%)**	
			**2015 Year**	**2016 Year**
**EC**	< 250	Excellent	Nil	Nil
	250–750	Good	93.3	93.3
	750–2250	Permissible	6.7	6.7
	>2250	Doubtful	Nil	Nil
**SAR**	0–10	Excellent	100	100
	10–18	Good	Nil	Nil
	18–26	Doubtful	Nil	Nil
	> 26	Unsuitable	Nil	Nil
**RSC**	< 1.25	Good	100	100
	1.25–2.5	Doubtful	Nil	Nil
	> 2.5	Unsuitable	Nil	Nil
**KR**	< 1	suitable	100	100
	1–2	Marginal suitable	Nil	Nil
	> 2	Unsuitable	Nil	Nil
**SSP**	< 50	Good	100	100
	> 50	Unsuitable	Nil	Nil
**PI**	> 75	Class-I	Nil	Nil
	25–75	Class-II	100	100
	< 25	Class-III	Nil	Nil
**MH**	< 50	Suitable	100	100
	> 50	Harmful &Unsuitable	Nil	Nil
**Na%**	< 20	Excellent	100	100
	20–40	Good	Nil	Nil
	40–60	Permissible	Nil	Nil
	60–80	Doubtful	Nil	Nil
	> 80	Unsuitable	Nil	Nil
**T.H**	< 75	Soft	Nil	Nil
	75–150	Moderately hard	Nil	Nil
	150–300	Hard	86.7	76.7
	> 300	Very hard	13.3	23.3

**Table 6:** Summary of water quality indices in present study.

**Indices**	**Formula**
Residual sodium carbonate (RSC)	${\text{RSC}=(\text{CO}}_{3}^{2-}+{\mathrm{HCO}}_{3}^{-})+\left({\mathrm{Ca}}^{2+}+{\mathrm{Mg}}^{2+}\right)$
Permeability index (PI)	$\mathrm{PI}=\frac{\mathrm{Na}+K+\sqrt{\mathrm{HCO}3}}{\mathrm{Ca}+\mathrm{Mg}+\mathrm{Na}+K}\times 100$
Kelly's ratio (KR)	$\mathrm{KR}=\frac{\mathrm{Na}}{\mathrm{Ca}+\mathrm{Mg}}$
Magnesium hazard(MH)	$\mathrm{MH}=\frac{\mathrm{Mg}}{\mathrm{Ca}+\mathrm{Mg}}\;\times 100\;$
Sodium percentage (Na %)	$\mathrm{Na}\%=\frac{\mathrm{Na}+K}{\mathrm{Ca}+\mathrm{Mg}+\mathrm{Na}+K}\;\times 100$
Sodium adsorption ratio (SAR)	$\mathrm{SAR}=\frac{\mathrm{Na}}{\sqrt{(\mathrm{Ca}+\mathrm{Mg})/2}}\times 100$
Soluble sodium percentage (SSP)	$\mathrm{SSP}=\frac{\mathrm{Na}}{\mathrm{Ca}+\mathrm{Mg}+\mathrm{Na}}\;\times 100\;$

**Table 7:** Pearson's correlation coefficient.

	pH	Na	Mg	Ca	HCO_3_	CL	SO_4_	TDS	EC	TH
pH	1									
Na	− 0.416**	1								
Mg	− 0.424**	0.30*	1.00							
Ca	− 0.451**	0.578**	0.544**	1.00						
HCO_3_	− 0.569**	0.551**	0.753**	0.884**	1					
CL	− 0.384**	0.820**	0.572**	0.749**	0.672**	1				
SO_4_	− 0.148	0.425**	0.591**	0.581**	0.389**	0.678**	1			
TDS	− 0.516**	0.641**	0.799**	0.924**	0.938**	0.829**	0.671**	1		
EC	− 0.551**	0.573**	0.695**	0.835**	0.895**	0.690**	0.462**	0.890**	1	
TH	− 0.499**	0.523**	0.836**	0.915**	0.940**	0.764**	0.663**	0.988**	0.880**	1

** Correlation is significant at the 0.01 level (2-tailed).

* Correlation is significant at the 0.05 level (2-tailed).

## 2 Experimental design, materials and methods

### 2.1 Description of study area

Sarpol-e Zahab city in Kermanshah province are located in west of Iran between the latitudes 34.4514 ° N and longitudes 45.8612 °E, encompassing an area of about 935.2 km^2^. Also the SarPol-e Zahab city has a cold and dry climate and the average altitude of the city is 550 m above sea level. It is worth noting that the average rainfall is 111 mm, with the minimum and maximum temperature of 1/1 ° C and 11.3 ° C, respectively.

### 2.2 Materials and methods

In order to assess the physico-chemical parameters, a total of 30 groundwater samples were collected from Sarpol-e Zahab city between years the of 2015 and 2016 (Fig. 1). Sampling was conducted with one‑liter polyethylene bottles which were immersed in nitric acid for 24 h then washed with 10% HCL and finally washed with distilled water. Before the samples were taken, sampling containers had been rinsed at least three times with water. Experiments have been done in two total categories of system tests and titrimetric tests including temporary and permanent hardness, calcium, magnesium and chloride. Also system tests including PH and electrical conductivity (EC) measured by PH meter device (pHwtw model) and Esi meter (wbw), respectively. The analysis of anions and cations of sulfate was also done by spectrophotometer Hatch (DR 5000 model) in water and wastewater laboratory of Kermanshah. Total hardness was determined by EDTA titrimetric method and TDS was measured gravimetrically [2], [3], [4], [5], [6], [7], [8], [9], [10].

Statistical analyses including Spearman correlation coefficients and factor analysis display good correlation between physicochemical parameters (EC, TDS and TH) and Na^+^, Mg^2+^, Ca^2+^, Cl^−^ as well as ${\mathrm{SO}}_{4}^{2-}$ ionic constituents of groundwater with SPSS (IBM Corp. Released 2016. IBM SPSS Statistics for Windows, Version 24.0. Armonk, NY: IBM Corp).

Finally, in order to understand chemical character of the groundwater and relationships between the dissolved ionic constituents, the hydrochemical data has been plotted on Piper diagram (Piper 1944) using AqQA software (Fig. 2).
